# Concomitant homozygosity for the prothrombin gene variant with mild deficiency of antithrombin III in a patient with multiple hepatic infarctions: a case report

**DOI:** 10.1186/1752-1947-4-122

**Published:** 2010-04-29

**Authors:** Theodore Emmanuelle, Belkys Husein, Javaid Iqbal, M Macheta, Peter Isaacs

**Affiliations:** 1 Department of Gastroenterology, Blackpool Victoria Hospital, Whinney Heys Road, Blackpool, Lancashire, UK; 2 Department of Haematology, Blackpool Victoria Hospital, Whinney Heys Road, Blackpool, Lancashire, UK

## Abstract

**Introduction:**

Hereditary causes of visceral thrombosis or thrombosis should be sought among young patients. We present a case of a young man presenting with multiple hepatic infarctions resulting in portal hypertension due to homozygosity of the prothrombin gene mutation not previously described in literature.

**Case presentation:**

A 42-year-old Caucasian man with a previous history of idiopathic deep vein thrombosis 11 years earlier presented with vague abdominal pains and mildly abnormal liver function tests. An ultrasound and computed tomography scan showed evidence of hepatic infarction and portal hypertension (splenic varices). A thrombophilia screen confirmed a homozygous mutation for the prothrombin gene mutation, with mildly reduced levels of anti-thrombin III (AT III). Subsequent testing of his father and brother revealed heterozygosity for the same gene mutation.

**Conclusion:**

Hepatic infarction is unusual due to the rich dual arterial and venous blood supply to the liver. In the absence of an arterial or haemodynamic insult causing hepatic infarction, a thrombophilia should be considered. To our knowledge, this is the first reported case of a hepatic infarction due to homozygosity of the prothrombin gene mutation. It is unclear whether homozygotes have a higher risk of thrombosis than heterozygotes. In someone presenting with a first thrombosis with this mutation, the case for life-long anticoagulation is unclear, but it may be necessary to prevent a second and more severe second thrombotic event, as occurred in this case.

## Introduction

Thrombophilia is an acquired or inherited tendency for recurrent thrombotic episodes. There is no obvious clinical reason for thrombosis. However, an inherited factor should be considered, especially in patients who have their first idiopathic venous thromboembolism before the age of 50, recurrent thrombotic episodes, or a first degree relative with thrombosis before the age of 50 [1].

Inherited thrombophilia can be due to inactive variants of endogenous anticoagulants (protein C, S and anti-thrombin III). Or, very rarely, it can be due to overactive variants of normal procoagulants, such as dysfibrinogenaemia and the relatively recently described prothrombin gene variant. Factor V Leiden is the most common variant, accounting for up to 40% of cases [2].

The prothrombin gene variant is a mutation (transition of guanine to adenine) at nucleotide 20210 of the prothrombin gene. Heterozygous carriers of this mutation have elevated plasma prothrombin and are prone to venous thrombotic episodes [3].

We describe the case of a 42-year-old man who presented with abdominal pain attributed to multiple hepatic infarctions who had a combination of homozygosity for the prothrombin gene variant and mild deficiency of antithrombin III (AT III).

## Case presentation

A 42-year-old Caucasian man presented with a one-year history of worsening vague abdominal pains over the past few months. The pain radiated to his back, and was associated with fever. He did not smoke and his alcohol consumption was 25 units per week.

At the age of 31 years, he had a right spontaneous lower limb deep vein thrombosis (DVT), with post-phlebitic leg swelling. His father had previously had two spontaneous DVTs, and his brother, aged 30, had a DVT following a herniorrhaphy.

An examination and investigations revealed a normal blood pressure and pulse. There was generalised upper abdominal tenderness, but no guarding or rebound. His cardiorespiratory examination was unremarkable. His blood tests revealed the following: bilirubin 21 μmol/L (normal range 5-17 μmol/L), aspartate aminotransferase (AST) 50 IU/L (normal range <40 IU/L), alkaline phosphatise (ALKP) 271 U/L (normal range <105 IU/L), gamma glutamyl transferase (GGT) 150 IU/L (normal range <35 IU/L), amylase 25 U/L, international normalised ratio (INR) 1.1, prothrombin time (PT) 27.4, C-reactive protein (CRP) 114 mg/L (normal range <4 mg/L), erythrocyte sedimentation rate (ESR) 53 mm/hr, normal full blood count (platelets 190 × 10^9^/L) and normal renal function.

There was no growth in the blood culture. Serology for Epstein-Barr virus, hepatitis A virus and hepatitis C virus was negative. An autoimmune screen was negative (rheumatoid arthritis, antinuclear antibody, anti-mitochondrial antibody, anti-liver kidney microsomes, anti-smooth muscle antibody). An ultrasound scan of the patient's abdomen showed mild splenomegaly with a trace of ascites.

During a follow-up in clinic two months later, he complained of increased lethargy with reduced appetite. A unilateral leg oedema was noticed. A gastroscopy was normal and a computed tomography (CT) scan confirmed splenomegaly (13.5 cm), though he had a normal liver and had no ascites. An ultrasound Doppler examination showed a chronic DVT with incomplete recanalisation. Repeat blood tests resulted in the following: ALT 38 IU/L, ALKP 77 IU/L, bilirubin 22 μmol/L, AST 27 IU/L, GGT 58 IU/L, thrombocytopenia (78 × 10^6^/mm^3^) and neutropenia (1.65 × 10^6^/mm^3^), ESR 5 mm/hr.

Six months later, the patient experienced persisting vague abdominal pain, thrombocytopenia and a mild abnormality of the liver function tests. A repeat liver ultrasound showed a new 3 cm echo-poor area in the periphery of segment VIII, consistent with an infarct.

A repeat CT scan six months later showed multiple infarcts in the liver, numerous dilated veins on the greater and lesser curves of his stomach, splenic hilum, and surrounding the portal vein, consistent with portal hypertension.

A thrombophilia screen for anticardiolipin antibodies, lupus anticoagulant, protein C and protein S deficiency, factor V Leiden mutation and paroxysmal nocturnal haemoglobinuria were all negative. He was found to be homozygous for the prothrombin G20210A mutation with mild deficiency for antithrombin III (AT III 73%, normal range 80%-120%). His father and brother were subsequently tested and found to be heterozygous for the prothrombin gene variant. He was started on life-long warfarin.

## Discussion

Prothrombin is encoded by a 21-kb-pair gene localised on chromosome 11. Poort *et al*. identified a transition (guanine to adenine) at nucleotide 20210 in the 3' untranslated region of the prothrombin gene as a risk factor for thrombosis [4]. This is possible because the variant prothrombin is resistant to degradation. Heterozygotes for the variant have 30% increased plasma prothrombin levels [4,5].

The level of thrombotic risk in homozygotes is not known as only a few cases have been reported [4,6,7], but their prothrombin levels are not always raised [8]. A young Mexican male homozygote suffered a myocardial infarction, ilio-femoral venous thrombosis and massive pulmonary embolism [9]. In contrast to this, two homozygotes had no thrombosis, suggesting that the mutation alone is less risky than other genetic risk factors [6].

Visceral vein thrombosis is rare but quite typical of inherited thrombophilia [9], especially mesenteric vein occlusion [10-13]. Heterozygotes have been reported with Budd-Chiari syndrome [10], hepatic vein thrombosis [12], or portal and mesenteric vein thrombosis [11,13]. But this is the first report of G20210A homozygosity for the prothrombin gene variant and mild deficiency of AT III affecting the liver.

A hepatic infarction is rare because of the richly anastamosing collateral arterial supply, and the dual blood supply from the portal vein and hepatic artery. In this case, there was portal hypertension as evidenced by splenomegaly, thrombocytopenia, and peri-gastric venous varices. The portal vein may have thrombosed and recanalised, similar to the patient's leg DVT and the hepatic infarct, which must have resulted from the interruption of both the arterial and venous blood supply, which is usually needed for such an injury in the absence of an established cirrhosis.

## Conclusion

Individuals who are heterozygotes for multiple prothrombotic mutations are prone to thromboses. But the decision to offer anticoagulation must be based on clinical manifestations. However, combinations of factor V Leiden and protein C deficiency, protein S deficiency or AT III deficiency greatly increase the risk of severe and recurrent thrombosis [14]. The four-year risk of recurrent DVT for 20210A heterozygotes is no greater than that for non-thrombophilic DVT patients [15,16]; but a 10-year follow-up study showed the risk to be increased 2.4 fold [17].

There is at present insufficient evidence pointing to a need for life-long anticoagulation of heterozygous carriers of the prothrombin gene mutation after a single episode of DVT. But in this case, there was limb DVT and a visceral thrombosis with secondary portal hypertension justifying life-long anticoagulation.

## Competing interests

The authors declare that they have no competing interests.

## Authors' contributions

All authors contributed in the preparation of this report.

## Consent

Written informed consent was obtained from the patient for publication of this case report and accompanying images. A copy of the written consent is available for review by the Editor-in-Chief of this journal.
